# Betaine addition as a potent ruminal fermentation modulator under hyperthermal and hyperosmotic conditions *in vitro*

**DOI:** 10.1002/jsfa.10255

**Published:** 2020-01-28

**Authors:** Mubarik Mahmood, Renée Maxine Petri, Ana Gavrău, Qendrim Zebeli, Ratchaneewan Khiaosa‐ard

**Affiliations:** ^1^ Department for Farm Animals and Veterinary Public Health Institute of Animal Nutrition and Functional Plant Compounds, University of Veterinary Medicine Vienna Vienna Austria; ^2^ Section of Animal Nutrition, Department of Animal Sciences University of Veterinary and Animal Sciences, Lahore sub‐campus Jhang Jhang Pakistan; ^3^ AGRANA Sales & Marketing GmbH Vienna Austria

**Keywords:** betaine, hyperosmotic stress, hyperthermal stress, ruminal fermentation, 16S rRNA bacteria

## Abstract

**BACKGROUND:**

Climatic and dietary shifts predispose ruminal microbes to hyperthermal and hyperosmotic stress, leading to poor fermentation and subsequently adverse effects on ruminant productivity. Betaine may function as substrate, osmolyte, antioxidant, and methyl donor for microbes. However, its effect depends on the extent of microbial catabolism. This study revealed the ruminal disappearance kinetics of betaine and its dose effect on ruminal fermentation during thermal and osmotic stress using a rumen simulation technique.

**RESULTS:**

Three different betaine doses were used: 0, 50, and 286 mg L^−1^; each was assigned to two incubation temperatures (39.5 and 42 °C) and two osmotic conditions (295 and 420 mOsmol kg^−1^). Betaine disappeared rapidly within the first 6 h of incubation; however, the rate was lower during hyperosmotic stress (*P* < 0.05), the stress condition that also suppressed the overall fermentation and degradation of organic nutrients and decreased the bacterial diversity (*P* < 0.001). During hyperosmotic stress, betaine shifted the fermentation pathway to more propionate (*P* < 0.05). Betaine counteracted the negative effect of hyperthermal stress on total short‐chain fatty acid concentration (*P* < 0.05) without affecting the composition. Both stress conditions shifted the bacterial composition, but the effect of betaine was minimal.

**CONCLUSION:**

Despite its rapid ruminal disappearance, betaine modulated microbial fermentation in different ways depending on stress conditions, indicating the plasticity of the betaine effect in response to various kinds of physicochemical stress. Although betaine did not affect the abundance of ruminal microbiota, the enhanced fermentation suggests an improved microbial metabolic activity under stress conditions. © 2020 The Authors. *Journal of The Science of Food and Agriculture* published by John Wiley & Sons Ltd on behalf of Society of Chemical Industry.

## INTRODUCTION

Ruminants are dependent upon ruminal microbes, which enable many biochemical functions, such as formation of short‐chain fatty acids (SCFAs) and microbial protein synthesis by fermenting feedstuffs that are typically not digested by the host.^1^ The SCFAs and microbial protein meet the majority of a ruminant's energy and protein demands. Within the rumen ecosystem, osmolality and temperature are important physicochemical parameters that impact the activity, survival, and balance among the ruminal microbial population.^2^, ^3^, ^4^ It has been shown that high‐concentrate feeding, which results in increased dry matter intake and is rapidly fermented, elevates the temperature and osmolality of the rumen content.^5^, ^6^, ^7^ An escalated environmental temperature alone could also raise ruminal temperature.^8^, ^9^ As a result of such physicochemical changes, the metabolism and growth of ruminal microbes are placed under stress, which, depending on the severity, can compromise the stability of the rumen ecosystem and thus animal health. In line with that, high‐grain feeding and heat stress exert a number of physiological challenges in cattle.^6^, ^10^ Since diet is the largest determining factor of rumen microbial stability,^11^ diet fortification would be the most promising way to rectify the effects of physicochemical challenges to ruminal microbes, improving ruminal fermentation and subsequent energy supply to the cow.

Betaine, or trimethylglycine, is found naturally in microbes, animals, and plants, and it is highly abundant in sugar beet.^12^ As a zwitterion, betaine holds intracellular water molecules against a concentration gradient;^13^ therefore, it exerts an osmoprotective effect on cells^14^, ^15^ and has been suggested to be an active substance given to animals exposed to osmotic stress‐related disorders.^16^ Betaine also acts as a thermoprotectant for bacterial cells,^17^ and it serves as a methyl donor^18^ and a direct substrate for ruminal microbes.^19^, ^20^ It can also stabilize native protein structure and prevent molecular disintegration.^21^, ^22^ These characteristics make betaine suitable to normalize microbial cell physiology perturbed during heat and osmotic stress. However, betaine is believed to be rapidly catabolized by ruminal microbes,^23^ though this seems to depend on ruminal conditions, as the use of basal diet can interfere with the degradation kinetics.^24^ Despite the fact that betaine‐rich feedstuffs like sugar beet pulp and wheat are commonly fed to cattle, we know very little about the effectiveness of betaine as a rumen modulator based on its bioavailability and mode of action under various ruminal conditions.

To fill this research gap, this study was designed with the goal to investigate ruminal disappearance kinetics of betaine and the dose‐dependent effects on rumen microbial composition and ruminal fermentation characteristics under hyperthermal and hyperosmotic conditions, using a rumen simulation technique (Rusitec). We hypothesized that increased temperature and hyperosmolality would reduce microbial fermentation and population diversity. However, more betaine would be utilized by ruminal microbes to maintain fermentation and diversity under stress conditions, and thus supplementing betaine could beneficially modulate rumen fermentation.

## MATERIALS AND METHODS

### Experimental design and treatments

The experiment was carried out using two Rusitec systems, forming a total of 12 experimental units used in each experimental run. The trial was performed with six experimental runs, resulting in *n* = 6 per individual treatment. Each experimental run lasted for 10 days, with the first 5 days specified as an adjustment for equilibration of the system followed by the last 5 days for sampling. The test conditions were arranged as a 2 × 2 × 3 factorial design including two temperatures (normal: 39.5 °C; hyperthermal: 42 °C), two osmotic conditions (physiological: target at ~295 mOsmol kg^−1^ and pH 6.6; hyperosmotic: target at ~420 mOsmol kg^−1^ and pH 6.0), and three target betaine concentrations in the incubation liquid at 0 (CON), 51 (LB) and 286 mg L^−1^ (HB). The betaine doses were prepared from betaine obtained from sugar beet molasses (containing 40% v/v betaine, Actibeet® L; AGRANA Stärke GmbH, Vienna, Austria). Target temperatures were maintained by thermostatically controlled water baths. The high temperature chosen (42.0 °C) was based on the maximum temperature observed in cattle exposed to mild heat stress conditions.^5^ The hyperosmolality chosen followed the maximal values *in vivo* in response to feeding.^25^ The target pH and osmolality were successfully achieved by means of diet (Table 1) and buffer (Table 2). Accordingly, fermenters with physiological conditions were infused with McDougall's buffer^26^ with slight modifications and fed a 50% concentrate diet, whereas those with low pH and hyperosmotic stress were treated with a 65% concentrate diet and a modified buffer (Table 2). There were changeovers among fermenters assigned to betaine and heat treatments so that the treatments were not associated with certain instruments. All diet ingredients were ground with a Wiley mill (Pulverisette 25/19; Fritsch GmbH, Idar‐Oberstein, Germany) to pass through a 6 mm sieve before use. Silages were dried (oven drying at 65 °C for 48 h) prior to grinding.

**Table 1:** Ingredient and chemical composition of diets for physiological and hyperosmotic conditions

	Composition (g kg^−1^ dry matter)	
Item	Physiological	Hyperosmotic
**Ingredient**		
Grass silage	250	200
Corn silage	150	70
Hay	100	80
Concentrate mixture^a^	430	560
Protein concentrate^b^	70	90
**Chemical composition**		
Organic matter	929	931
Crude protein	165	165
Ash	71	69
Neutral detergent fiber	295	258
Ether extract	27	29
Non‐fiber carbohydrates^c^	442	478

a On dry matter basis (g kg^−1^): 216 barley; 216 wheat; 517 maize; and 52 vitamin and mineral supplement (Rindavit TMR 11 ASS‐CO + ATG; H. Wilhelm Schaumann GmbH & Co KG, Brunn/Gebirge, Austria).

b Rindastar 39 XP (H. Wilhelm Schaumann GmbH & Co KG).

c Non‐fiber carbohydrates = 1000 − (ash + crude protein + neutral detergent fiber + ether extract).

**Table 2:** Composition of buffers used for physiological and hyperosmotic stress conditions

	Concentration (mmol L^−1^)	
Component	Physiological buffer	Hyperosmotic buffer
NaHCO_3_	95.1	58.2
Na_2_HPO_4_·2H_2_O	23.6	13.1
NaCl	8.04	123.2
KCl	7.64	7.64
CaCl_2_·2H_2_O	0.37	0.37
MgCl_2_·6H_2_O	0.63	0.63

### Rusitec procedure

On the first day of each experimental run, the ruminal fluid and solid digesta were collected from three fistulated non‐lactating Holstein cows kept at the dairy research station (VetFarm) of Vetmeduni, Vienna. The donor cows were fed primarily hay and grass silage and cared for according to the Austrian guidelines of animal welfare.^27^ Collected ruminal fluid was pooled equally for all three cows after passing through four layers of medical gauze of 1 mm pore size. Subsequently, each vessel was inoculated with 100 mL of the respective buffer and 600 mL of strained ruminal fluid. Two nylon bags (120 mm × 65 mm, 150 μm pore size; Fa. Linker Industrie‐Technik GmbH, Kassel, Germany), one filled with pooled solid digesta and another with the respective diet containing approximately 12 g diet dry matter (Table 1) were incubated in each fermenter. On the next day, the bag with solid digesta was replaced with a new bag containing the respective diet. During the entire experimental run, the flow of salivary buffer was regulated with the help of a 12‐channel peristaltic pump (model ISM932, Ismatec; Idex Health & Science GmbH, Wertheim, Germany) at a rate of 393 ± 17 mL per day. The outflow was collected in effluent bottles kept cool at 1 °C in a refrigerator.

For daily feed bag exchange, the feed bag incubated for 48 h was removed and replaced with a fresh feed bag. Before removal, the incubated feed bag was rinsed and squeezed with 40 mL of the pre‐warmed respective buffer. The effluent bottle was emptied and the gas‐tight bag changed daily. After feed bag exchange, the fermenter was closed and flushed with nitrogen gas for 3 min to re‐establish anaerobic conditions. Shortly afterward, betaine was thoroughly dosed into the fermenter via the valve.

### Daily measurements and laboratory analyses

During the sampling period (d6–d10) the 48 h incubated feed bags were washed with cold water in a tub until the water became clear and then stored at −20 °C for later analysis of dry matter, organic matter (OM), crude protein, crude fat, and neutral detergent fiber (NDF) according to the method of VDLUFA.^28^ The composition of nutrients in feed, before and after incubation, was used for estimation of apparent nutrient disappearance.

The fermenter fluid was collected daily (d6–d10) from each fermenter to measure ruminal fermentation characteristics as described previously.^29^ Accordingly, the incubation liquid was immediately measured for pH and redox potential using a pH meter (Seven Multi TM; Mettler‐Toledo GmbH, Schwerzenbach, Switzerland) equipped with separate electrodes (InLab Expert Pro‐ISM for pH and Pt4805‐DPA‐SC‐S8/120 for redox; Mettler‐Toledo GmbH). Another portion of the aliquot was preserved at −20 °C for later analyses including SCFAs using gas chromatography,^29^ ammonia using the indophenol reaction method,^30^ and osmolality using a multisample osmometer (Osmo Pro®; WMDE B.V., Bergerweg, Netherlands). For betaine concentration analysis, on d7, there were interval samplings of fluid from all fermenters at 0, 1, 2, 4, 6, and 24 h after betaine dosing to determine betaine disappearance kinetics. Quantification of betaine in the fermenter fluid was done using high‐performance liquid chromatography with refractive index detection and equipped with a 300 mm × 7.8 mm column (Aminex HPX 87‐K; Bio‐Rad, Hercules, CA, USA). Briefly, samples were thawed and centrifuged at room temperature and 12 045×*g* for 15 min. The samples were then filtered with a 0.45 μm filter and the clear solution was injected into high‐performance liquid chromatograph without further dilution. Separation of the betaine was done on a Bio‐Rad Aminex HPX 87‐K column. The injection volume was kept at 20 μL, and the oven temperature was fixed at 65 °C. Afterward, the separation was performed under isocratic elution conditions using a mobile phase containing 50 mmol L^−1^ dipotassium hydrogenphosphate and 50 mmol L^−1^ monopotassium phosphate. The mobile phase was delivered at a flow rate of 0.6 mL min^−1^, and quantification was based on refractive index detection.

### DNA extraction, sequencing, sequence processing, and bioinformatics analysis

On d10, fermenter fluid was collected and snap‐frozen in liquid nitrogen, and solid samples were collected and all samples stored at −20 °C for microbial analysis. Total microbial DNA was extracted from about 800 μL of fluid and 0.25 g of solid digesta using the DNeasy PowerSoil Kit (Qiagen, Hilden, Germany) following the method described previously,^31^ with some modifications. In brief, samples were treated with heat (incubation at 95 °C for 5 min), chemical (100 μL of 100 mg mL^−1^ lysozyme; 10 μL of 2.5 U mL^−1^ mutanolysin (Sigma Aldrich, Vienna, Austria), 21 μL of 18.6 mg mL^−1^ proteinase K (Sigma Aldrich) and mechanical disruption (bead‐beating using the FastPrep‐24 instrument (MP Biomedical, Santa Ana, CA, USA)) to improve DNA efficiency, according to previously published procedures.^32^ DNA concentration was determined by a Qubit 2.0 fluorometer (Life Technologies, Carlsbad, CA, USA) and the Qubit double‐stranded DNA (dsDNA) HS Assay Kit (Life Technologies). From each DNA sample, the same concentration of liquid and solid digesta fractions were pooled to a total volume of 40 μL and sent for amplicon sequencing using Illumina MiSeq paired‐ends sequencing technology (Microsynth AG, Balgach, Switzerland). Targeted amplification of V3–V5 of the bacterial 16S rRNA gene was performed, using the primer set 357F (5′‐CCTACGGGAGGCAGCAG‐3′) and 926R (5′‐CCGTCAATTCMTTTRAGT‐3′).^33^ The sequencing procedure was as described in Bagheri *et al*.^31^ Data quality control and analyses were performed using the open‐source QIIME pipeline.^34^ The 7 581 334 raw reads were quality filtered based on length (415 bp) and quality of the reads. Screening for chimeric sequences was done using USEARCH,^35^ then aligned and clustered using PyNAST^36^ and the SILVA‐128 database.^37^ The degree of similarity between sequences was defined as 97% to obtain operational taxonomic unit (OTU) identity at the genus level. OTUs that clustered with less than ten reads were manually removed. These processes resulted in 7 279 716 reads in 72 samples with a mean of 93 329 reads per sample. These reads could be clustered into 4453 unique OTUs with a minimum of ten sequences per OTU. 872 OTUs had a relative abundance of >0.01%, resulting in 3581 rare OTUs. For alpha diversity analysis, Chao1, Shannon, and Simpson indices were used. Sequencing data are available in the BioProject SRA database under accession number PRJNA558251.

### Statistical analysis

Statistical analyses were performed using the MIXED procedure of SAS (version 9.4; SAS Institute Inc., Cary, NC, USA). The statistical model included betaine supplementation, incubation temperature, and osmolality along with their two‐way and three‐way interactions. The variation between experimental runs was considered as a random effect, and data for the daily measurements of the same fermenter were analyzed as a repeated measure with compound symmetry as the variance–covariance structure. Only for betaine added groups was the disappearance kinetics of betaine fitted as an exponential decay function (*y* = *a*e^*bx*^) using the NLIN procedure of SAS (version 9.4; SAS Institute Inc.) to estimate the kinetics parameters (*a* and *b*). Subsequently, each parameter was statistically analyzed using the MIXED procedure of SAS for the same main effects in the model as mentioned earlier. Correlation analysis was performed using the CORR procedure to obtain Pearson correlation coefficients. Mean values reported are least square means plus/minus the pooled standard error of the mean (SEM). Significance was declared at *P* ≤ 0.05 and a tendency of an effect at 0.05 < *P* ≤ 0.10.

## RESULTS

### Betaine degradation kinetics

As analyzed, the concentration of betaine in the incubation liquid at 0 h was 56 ± 6 mg L^−1^ (mean plus/minus standard deviation) for LB and 314 ± 41 mg L^−1^ for HB. The 0 h concentration of betaine in the CON group was 0.8 ± 1.2 mg L^−1^.

**Figure 1:**
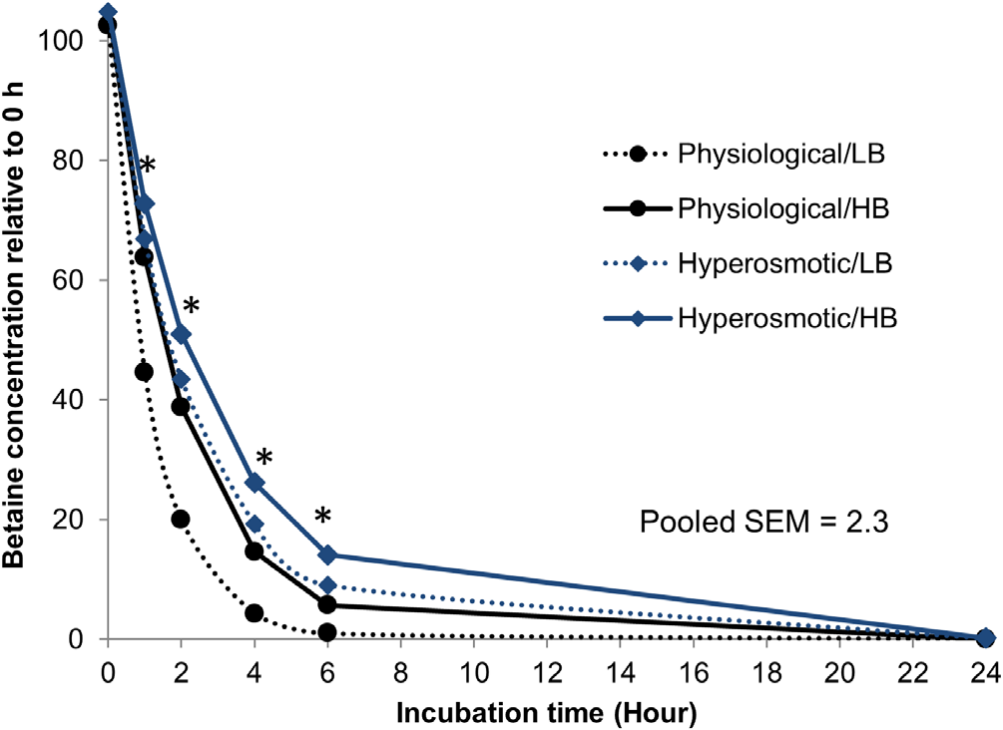
Ruminal disappearance of betaine (expressed as proportional change (%) of 0 h concentration) incubated under physiological and hyperosmotic rumen conditions *in vitro*. Target 0 h concentrations were 51 mg L^−1^ (LB) and 286 mg L^−1^ (HB). Asterisks indicate differences among treatments at each incubation hour (*P* < 0.05).

Betaine rapidly disappeared in the first 6 h after addition; however, the disappearance rate was dose dependent and was affected by the osmolality of fermenter fluid, but not by the incubation temperature (Fig. 1, Table 3). The highest disappearance rate *b* was found with LB under the physiological condition, whereas the lowest *b* was found with HB with the hyperosmotic condition (*P* < 0.05). The HB group incubated under the hyperosmotic condition maintained a higher betaine concentration than the other groups did (*P* < 0.05) during the first 6 h after addition. No betaine could be detected at 24 h after addition.

**Table 3:** Estimated parameters for the *in vitro* ruminal disappearance kinetics (*y* = *a*e^*bx*^) of betaine at different doses incubated under different incubation temperature and osmolality

Parameter	Betaine dose^a^	Physiological^b^		Hyperosmotic^b^		SEM	*P <* 0.05^c^
		39.5 °C	42 °C	39.5 °C	42 °C		
*a*	Low	103	102	104	106	0.99	B
	High	105	106	105	105		
*b*	Low	−0.80	−0.90	−0.44	−0.48	0.06	B, O, B × O
	High	−0.52	−0.50	−0.41	−0.35		

SEM: standard error of the mean.

a Target low dose: 51 mg L^−1^; target high dose: 286 mg L^−1^.

b Target osmolality of the physiological condition was 295 mOsmol kg^−1^ and the hyperosmotic condition 420 mOsmol kg^−1^.

c Only significant effects are listed; B: effect of betaine dose; O: effect of the osmolality of fermenter fluid.

### Ruminal fermentation

Table 4 displays the fermentation characteristics as affected by incubation conditions and betaine addition. Regardless of incubation condition, HB increased the osmolality and ammonia concentration compared with CON and LB (*P* < 0.05). Total SCFAs concentration per OM degraded was higher in LB and HB than in CON (*P* < 0.05).

**Table 4:** Ruminal fermentation characteristics as affected by incubation temperature, osmolality, betaine supplementation, and their interactions

Item	Temperature (T)		Osmolality (O)		Betaine (B)^a^			SEM	*P* value			
	39.5 °C	42 °C	Physiological	Hyperosmotic	CON	LB	HB		T	O	B	Interaction^b^
Osmolality (mOsmol kg^−1^)	414	414	347	481	411^b^	412^b^	420^a^	7	0.767	<0.001	<0.001	
pH	6.17	6.18	6.50	5.85	6.18	6.18	6.17	0.08	0.457	<0.001	0.546	(T × O × B)
Redox (mV)	−167	−163	−185	−146	−166	−164	−166	7	0.069	<0.001	0.860	
Ammonia (mmol L^−1^)	12.3	12.4	12.7	12.0	11.3^b^	11.7^b^	14.1^a^	1.4	0.492	0.000	<0.001	T × O
SCFAs (mmol L^−1^)	108	107	112	103	104	109	110	5	0.612	<0.001	<0.001	(T × B)
SCFAs (mmol L^−1^ g^−1^ OM degraded)	17.4	17.3	17.6	17.0	16.7^b^	17.5^a^	17.8^a^	1.1	0.790	0.030	0.004	
SCFAs (mol per 100 mol)												
Acetate	42.9	44.8	45.8	41.9	43.2	43.7	44.6	1.5	<0.001	<0.001	0.003	T × O, O × B
Propionate	27.9	24.7	25.0	27.6	25.9	26.7	26.3	1.5	<0.001	0.005	0.449	(O × B), (T × O × B)
Butyrate	17.1	17.1	16.2	18.0	17.3	17.0	16.9	1.6	0.907	0.001	0.443	T × O
Isobutyrate	0.78	0.75	0.83	0.69	0.76	0.77	0.76	0.04	<0.001	<0.001	0.373	T × O × B
Valerate	4.51	5.23	5.02	4.72	5.15	4.84	4.62	0.54	<0.001	0.268	0.021	T × B, T × O × B
Isovalerate	4.22	4.37	4.20	4.39	4.45	4.22	4.22	0.20	0.018	0.058	0.002	T × B
Caproate	2.53	3.08	2.96	2.65	3.18^a^	2.72^ab^	2.51^b^	0.66	0.009	0.454	0.033	
Acetate to propionate	1.7	2.0	2.0	1.7	1.8	1.8	1.8	0.2	<0.001	0.005	0.875	T × O, T × B, O × B

SEM: standard error of the mean; SCFAs: short‐chain fatty acids; OM: organic matter.

Significant differences among the betaine groups (*P* < 0.05) are marked with superscripts when no interactions between betaine addition and other factors were detected.

a Target betaine concentration (mg L^−1^): CON, 0; LB, 51; HB, 286.

b Only effects with significance (*P* ≤ 0.05) or tendency marked with parentheses (*P* ≤ 0.10) are listed.

**Figure 2:**
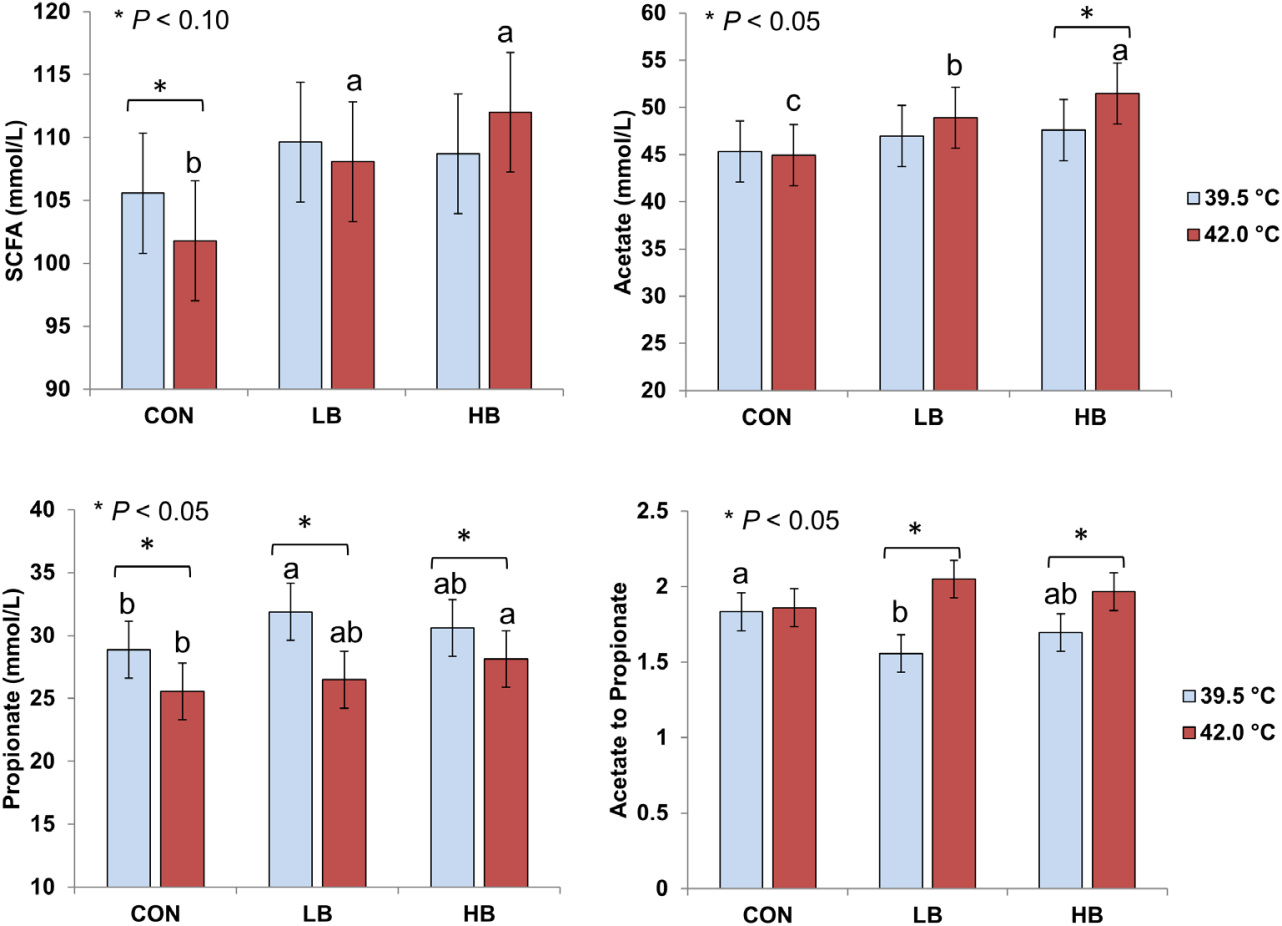
Concentrations of short‐chain fatty acids (SCFAs) as affected by betaine addition and incubation temperature. Least‐square means of betaine groups within each temperature condition sharing no common lower‐case letter differ at *P* < 0.05. Asterisks indicate differences between the temperature conditions within each betaine group (*P* < 0.05). CON: 0 mg L^−1^; LB: 51 mg L^−1^; HB: 286 mg L^−1^.

Interaction between betaine addition and incubation temperature tended to be significant for total SCFAs concentration (*P* = 0.087), acetate concentration (*P* = 0.067), and the molar percentage of isovalerate (0.022) (Table 4). As shown in Fig. 2, with the 39.5 °C incubation, betaine addition increased propionate concentration, thereby decreasing acetate to propionate ratio in LB compared with CON (*P* < 0.05), whereas HB showed intermediate values. Without betaine, the hyperthermal condition decreased concentrations of total SCFAs (*P* < 0.10) and propionate (*P* < 0.05), but LB and HB reversed the effect of hyperthermal condition on total SCFAs.

**Figure 3:**
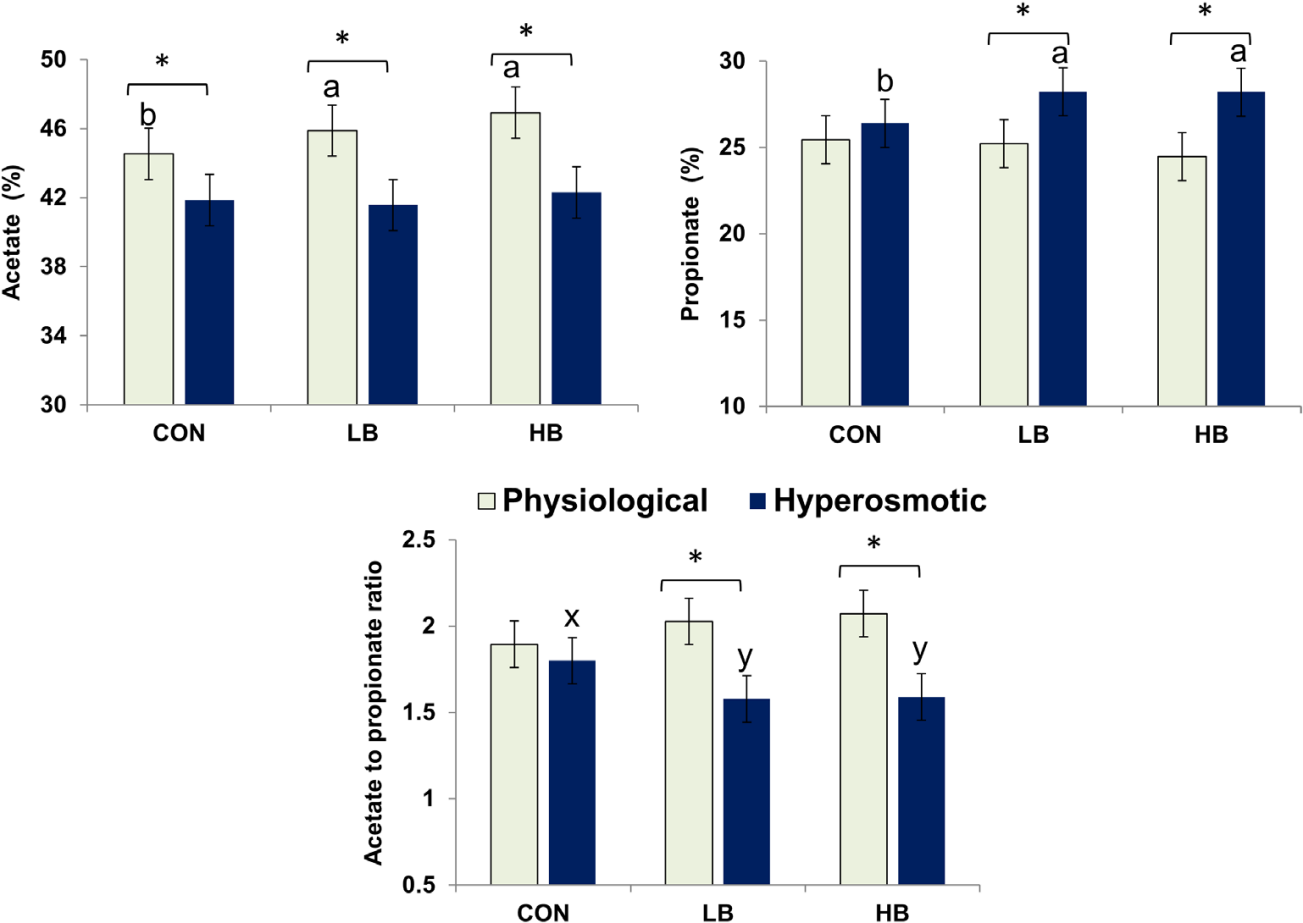
The composition of major short‐chain fatty acids as affected by betaine addition and osmolality of fermenter fluid. Least‐square means of betaine groups within each osmotic condition sharing no common lower‐case letter differ at *P* ≤ 0.05 (a, b) or 0.5 < *P* ≤ 0.10 (x, y). Asterisks indicate differences between the osmotic conditions within each betaine group (*P* < 0.05). CON: 0 mg L^−1^; LB: 51 mg L^−1^; HB: 286 mg L^−1^.

Overall, the hyperosmotic condition decreased overall fermentation, as seen by lowered SCFAs concentration compared with those under the physiological condition (*P <* 0.01). There was an osmolality × betaine interaction for molar percentages of acetate (*P* = 0.047) and propionate (*P* = 0.076) and acetate to propionate ratio (*P* = 0.045) (Table 4, Fig. 3). Without betaine, the hyperosmotic condition decreased acetate percentage compared with that of physiological condition (*P* < 0.05). Both LB and HB increased acetate percentage compared with CON (*P* < 0.05) under the physiological condition and promoted propionate percentage under the hyperosmotic stress (*P* < 0.05).

### Nutrient disappearance

Betaine addition did not affect the nutrient disappearances, except for a tendency for betaine × osmolality interaction for crude protein and fat disappearances (Table 5). The hyperosmotic condition reduced the disappearance of almost all nutrients studied especially NDF (−40%), but less so for non‐fiber carbohydrates (NFCs; −10%), but increased ash disappearance compared with that found under the physiological condition (*P* < 0.05). The hyperthermal condition increased the disappearance of NDF at the expense of NFCs disappearance (*P* < 0.05).

**Table 5:** Ruminal nutrient disappearance (percentage of supply) as affected by incubation temperature, osmolality, betaine supplementation, and their interactions (*n* = 5)

Item	Temperature (T)		Osmolality (O)		Betaine (B)^a^			SEM	*P* value			
	39.5 °C	42 °C	Physiological	Hyperosmotic	CON	LB	HB		T	O	B	Interaction^b^
Dry matter	57.7	58.1	59.2	56.6	58.0	57.9	57.8	0.8	0.281	<0.001	0.891	
Organic matter	56.0	56.4	57.7	54.7	56.3	56.1	56.2	0.8	0.262	<0.001	0.917	
Ash	80.7	80.6	79.7	81.5	80.8	80.7	80.4	1.0	0.814	<0.001	0.670	
Crude protein	70.0	70.1	71.5	68.6	69.8	70.8	69.5	1.0	0.977	<0.001	0.109	(O × B)
Neutral detergent fiber	10.1	13.0	14.2	8.9	12.4	11.0	11.3	2.4	<0.001	<0.001	0.265	
Non‐fiber carbohydrates	81.4	78.3	83.9	75.9	78.9	80.2	80.5	2.6	0.041	<0.001	0.650	
Ether extract	40.2	40.5	43.4	37.3	42.1	40.9	38.1	3.9	0.864	<0.001	0.087	T × O, (O × B)

SEM: standard error of the mean.

a Target betaine concentration (mg L^−1^): CON, 0; LB, 51; HB, 286.

b Only effects with significance (*P* ≤ 0.05) or tendency marked with parentheses (*P* ≤ 0.10) are listed.

### Bacterial diversity and composition

All microbial diversity indices including Chao1, Shannon, and Simpson indices were lower in the hyperosmotic condition than in physiological conditions (*P* < 0.001), whereas temperature and betaine addition did not affect the diversity.

**Figure 4:**
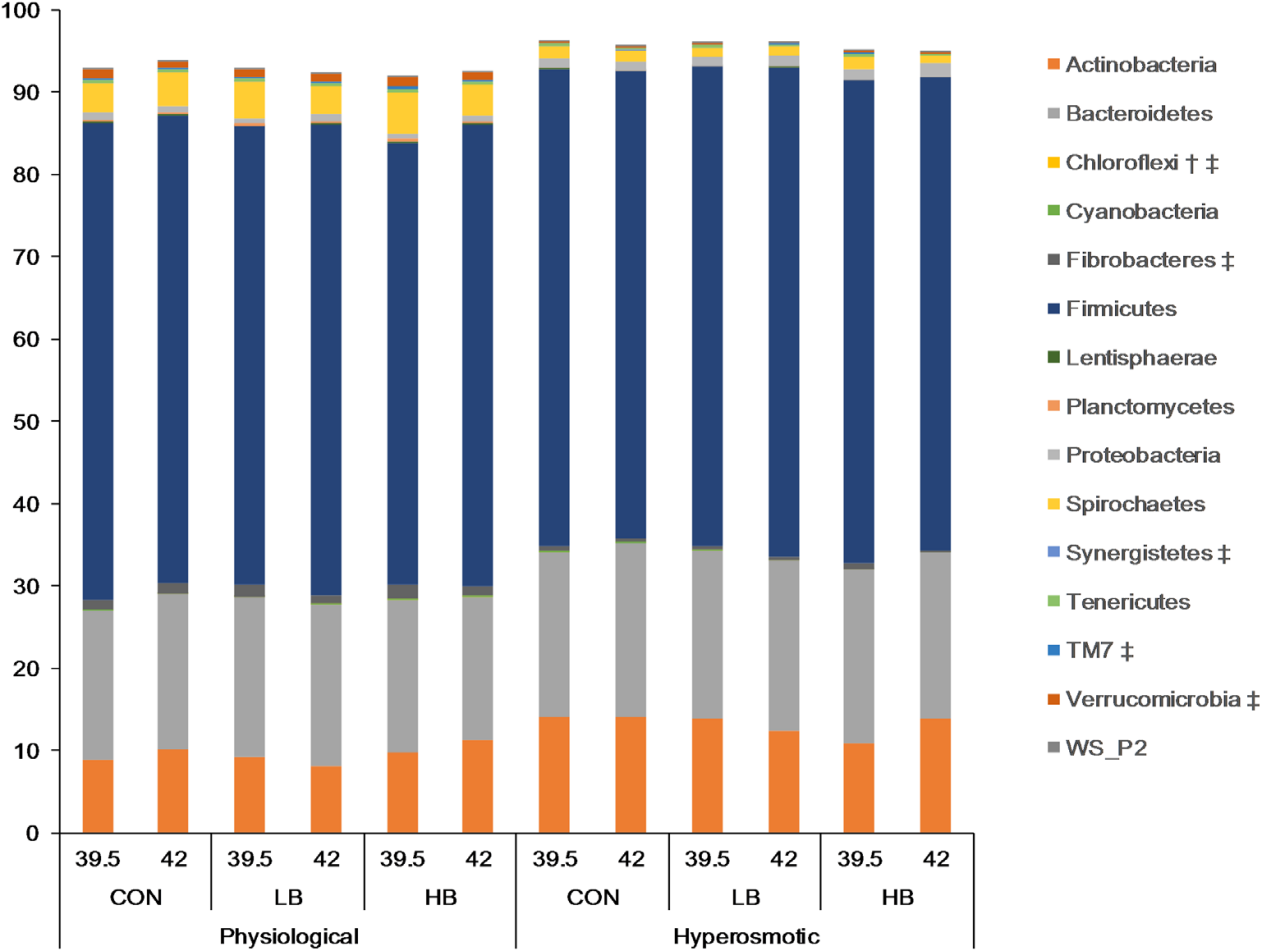
Relative abundances at the phylum level of pooled DNA from liquid and solid fermenter contents as affected by betaine concentration (CON: 0 mg L^−1^; LB: 5 mg L^−1^; HB: 286 mg L^−1^), incubation temperature (39.5 and 42.0 °C), and osmolality (physiological and hyperosmotic). Except for *Cyanobacteria* and *Firmicutes* (*P ≤* 0.10), the phyla show a significant effect of osmolality (*P* ≤ 0.01). ^†^Affected by betaine supplementation (*P* ≤ 0.01), ^‡^ Affected by temperature (*P* < 0.05).

*Firmicutes*, *Bacteroidetes*, and *Actinobacteria* were the three most abundant phyla (Fig. 4). Osmolality affected all microbial phyla (*P ≤* 0.01), with the exception of *Firmicutes* and *Cyanobacteria* (*P* ≤ 0.10). The hyperosmotic condition increased the relative abundance of the three dominant phyla, and *Proteobacteria*, whereas it decreased the abundance of the other minor phyla. The hyperthermal condition decreased the relative abundance of the phyla *Fibrobacteres*, *Synergistetes*, TM7, and *Verrucomicrobia* (*P <* 0.05). Of the identified phyla, betaine affected the relative abundance of *Chloroflexi* depending on the osmolality.

**Figure 5:**
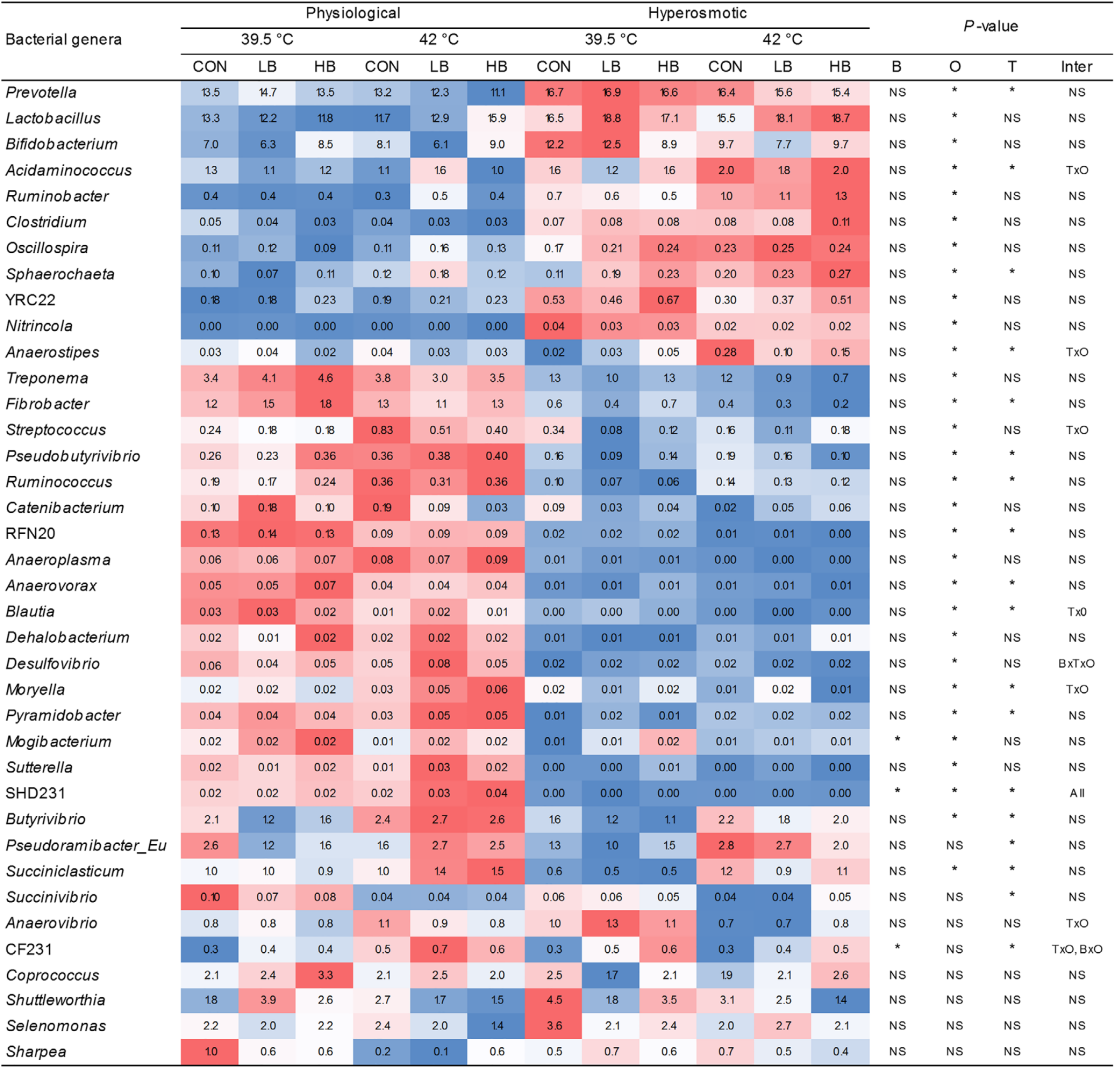
Relative abundances of microbial genera of pooled DNA from liquid and solid fermenter contents as affected by betaine concentration (CON: 0 mg L^−1^; LB: 5 mg L^−1^; HB: 286 mg L^−1^), incubation temperature (39.5 and 42.0 °C), and osmolality (physiological and hyperosmotic). B: betaine; T: temperature; O: osmolality; Inter: interaction; *P ≤ 0.05; All: all two‐way and three‐way interactions; NS: non‐significant. For each bacterial genus, the color system represents different microbial abundances among treatments: red and blue shades represent the upper half and the lower half values respectively; the darker the gradient, the higher the abundance.

The relative abundance of microbial genera was mainly affected by osmolality and temperature (*P* ≤ 0.05), whereas betaine affected only three genera of low abundances, including CF231, SHD231, and *Mogibacterium* (Fig. 5). Except for *Mogibacterium*, the betaine effect depended on the incubation condition. Of these three genera, only the genus SHD231 was positively correlated with propionate (*r* = −0.45, *P* < 0.05, detailed data not shown). *Prevotella*, *Lactobacillus*, and *Bifidobacterium*, which were the microbial genera with high abundances, increased under the hyperosmotic condition. Nineteen minor genera were sensitive and nine other minor genera were resistant to the hyperosmolality. The hyperthermal condition increased the relative abundance of *Butyrivibrio*, *Pseudoramibacter_Eubacterium*, and *Succiniclasticum* but decreased *Succinivibrio* and CF231.

## DISCUSSION

The addition of betaine in the rumen could be of high significance to the animal because betaine, as a substrate and methyl donor for microbes, directly modulates ruminal fermentation^23^, ^38^ and, as a compatible osmolyte, betaine may enable the microbial tolerance to stressors.^39^ Furthermore, ruminal metabolism of betaine directly influences its post ruminal effects such as methionine sparing and lipotropic effects.^12^, ^40^ The present study characterized ruminal disappearance kinetics of betaine and revealed how betaine modulates ruminal fermentation under influences of high temperature and osmolality, which are typical stressors caused by feeding as well as an escalated ambient temperature.^7^, ^8^, ^9^, ^41^

### Ruminal metabolism of betaine

In the present Rusitec experiment, we observed a rapid disappearance of betaine. Under physiological conditions in terms of osmolality and pH, about 5–15% remained at 4 h, with more betaine being recovered when administered at the high dose. By using [1‐^14^C]‑ and [methyl‐^14^C]‐labeled betaine, Mitchell *et al*.^23^ reported similar disappearance kinetics in the rumen of a sheep *in vivo*. Much slower disappearance rates were found in studies using a batch culture technique and testing very high betaine doses (>500 mg L^−1^).^24^, ^40^ This suggests either the limited capacity of ruminal microbes to handle large amounts of betaine or that the disappearance from the rumen is largely due to the flow of liquid to the lower gut, considering that betaine is water soluble. However, based on our experimental setup, the effluent outflow was relatively much slower than the disappearance rate. Furthermore, similar to other studies,^42^, ^43^ we also found a concomitant increase in acetate and ammonia concentrations, which are products of the microbial catabolism of betaine.^19^, ^23^ Therefore, the observed disappearance was largely a result of microbial activities. Notably, because we measured free betaine in the *in vitro* liquid, another source of betaine disappearance could be attributed to the incorporation by microbial cells in order to maintain water and ionic balances.^44^

We showed for the first time that the extensive disappearance of rumen‐unprotected betaine could be delayed by hyperosmolality, whereas hyperthermal stress had no effect. The findings, however, did not reflect our hypothesis that more betaine would be incorporated into microbial cells, which will enable them to adapt themselves to fluctuations in external osmotic pressures.^44^, ^45^, ^46^ Bacterial accumulation of betaine has been shown to be increased in response to hyperosmolality;^47^, ^48^ still, the required amounts seem small.^48^ Again, this hints that betaine disappearance appeared to be largely dictated by betaine catabolism in the present study. As suggested by Löest *et al*.,^24^ betaine catabolism is linked to overall fermentation. Consistently, in our study, the hyperosmotic condition, which stalled overall microbial fermentation, decreased betaine disappearance. Despite some alterations in betaine disappearance rate based on hyperosmolality and supplementation dosage, ruminal microbiota were capable of metabolizing large amounts of betaine, and no betaine was recovered in the fermenters after 24 h. A similar time threshold was reported in a batch culture study using a rumen‐unprotected betaine source.^24^

### Effects of betaine on ruminal fermentation in response to the stressors

Betaine acts as a thermoprotectant^17^ and as an osmoprotectant^47^ for bacterial cells and is a direct substrate of bacteria.^19^ Therefore, we expected beneficial effects of betaine supplementation on ruminal fermentation, especially when ruminal microbes are exposed to physicochemical stress. Our *in vitro* data showed that the hyperthermal stress suppressed ruminal fermentation, which was mainly attributed to decreased propionate production. This explains previous indications that heat stress alters carbohydrate metabolism, leading to lower plasma glucose and lactose secretion in the milk.^49^ In line with decreased propionate, the disappearance of NFCs was also lowered by the hyperthermal stress, and our data on microbial abundances suggest heat sensitivity of some starch‐degrading microbes like the genus *Succinivibrio*.^50^ On the contrary, the hyperthermal stress promoted NDF degradation despite the decreased abundance of the genus *Fibrobactor*, which was consistent with a previous study in cattle.^51^ However, another cellulolytic genus, *Ruminococcus*,^52^ thrived in the hyperthermal condition. Besides the abundance, changes in the activity of the existing microbes cannot be ruled out. Interestingly, the negative effect of hyperthermal stress on fermentation was overcome by betaine, as indicated by increased SCFAs production, including the concentrations adjusted for degraded OM. The positive effect of betaine supplementation was likely due to its thermoprotective effects^17^ rather than its direct effect on the microbial abundance and composition. Notably, both acetate and propionate production increased by betaine under the hyperthermal condition, although propionate is not a catabolic product of betaine degradation. Therefore, the improved fermentation under hyperthermal stress was not only a result of more betaine given to the microbes but also because betaine promoted microbial carbohydrate fermentation. This theory is supported by production data from a study^22^ that reported 1.5 kg day^−1^ more milk production in mid‐lactation Holstein cows when betaine was supplemented in the diet during hot months of the year.

The presence of betaine could not reverse the negative effect of hyperosmolality on suppressing fermentation; instead, betaine shifted the fermentation pathways. The increased molar percentage of acetate with betaine addition was evident under normal osmolality. With elevated osmolalities above 400 mOsmol kg^−1^, resembling the postprandial range *in vivo*,^3^ betaine addition increased the propionate percentage, although it did not impact the microbial community composition. Our correlation analysis indicates that SHD231 contributed to propionate percentage, but this genus almost vanished under the hyperosmotic condition and cannot explain the propionate‐promoting effect of betaine. A study by Rink *et al*.^53^ reported similar results regarding increased propionate production in the hindgut during hyperosmotic stress following betaine supplementation in the diet of pigs. According to Forbes and Barrio,^54^ cellulose degradation is suppressed at osmolalities above 400 mOsmol kg^−1^. In our study, the major fibrolytic bacteria (including the genera *Fibrobactor*, *Ruminococcus*, and *Trepanoma*) were sensitive to hyperosmolality, whereas amylolytic bacteria, propionate producers, and acid‐tolerant bacteria (such as *Prevotella*, *Lactobacillus*, and *Bifidobacterium*) proliferated, a characteristic that has been previously associated with high‐grain feeding.^55^, ^56^ Consistently, we found that NDF degradation was suppressed to a greater extent by hyperosmolality than the degradation of NFCs was. The further increase in propionate percentage upon betaine addition under the hyperosmotic stress suggests that betaine is used by the dominating microbes to support fermentation.

## CONCLUSION

Betaine was rapidly degraded by ruminal microbiota *in vitro*, but the degradation was delayed during hyperosmotic stress, the stress condition that also largely compromised overall degradation of major nutrients and shifted the fermentation pathway to more propionate. Without affecting the microbial composition and diversity, betaine supplementation altered their functionality and, therefore, promoted ruminal fermentation manifested by the dominant microbial community. In a nutshell, ruminal microbiota can genuinely benefit from betaine to support their metabolism and growth.
